# Clinical experiences with 6-monthly paliperidone palmitate after 12 months of use. A retrospective study

**DOI:** 10.1192/j.eurpsy.2023.1043

**Published:** 2023-07-19

**Authors:** S. Benavente López, A. Parra González, S. Bolaño Mendoza, A. Lara Fernández, A. Herencias Nevado, E. Baca García

**Affiliations:** Psychiatry, Hospital Universitario Infanta Elena, Madrid, Spain

## Abstract

**Introduction:**

Long-acting injectable antipsychotics (LAIA) have provided a significant improvement in the treatment of schizophrenia. Although there is already significant clinical experience with paliperidone palmitate, it is important to evaluate the clinical response of patients to this new 6-monthly presentation, so descriptive studies based on real clinical evidence can be very useful for this purpose.

**Objectives:**

The main objective of the study is to describe the use of 6-monthly paliperidone palmitate in routine clinical practice, providing variables that objectify the evolution such as the number of admissions and visits to the emergency room.

**Methods:**

Retrospective descriptive study with a sample selected by non-probabilistic consecutive sampling, retrospective type, in a time interval of 12 months (n=40). The patients selected were all those who received 6-monthly paliperidone palmitate treatment, with a diagnosis of schizophrenia, in 12 months of use at Hospital Universitario Infanta Elena. A descriptive analysis was performed. Mean and standard deviation were calculated for quantitative variables and N and percentage for categorical variables.

**Results:**

A total of 40 administrations of 6-monthly paliperidone palmitate were performed in the study. None of the patients presented adverse reactions related to the administration of the drug, not reporting local pain or inflammation of the puncture area, except for the characteristic discomfort of an intramuscular puncture. Regarding the efficacy of 6-monthly paliperidone palmitate, none of the patients presented a psychotic decompensation after its administration, maintaining psychopathological stability after the change. The switch to 6-monthly paliperidone palmitate was made from both 1-monthly paliperidone palmitate and 3-monthly paliperidone palmitate, both showing the same efficacy. Regarding tolerability, all the patients who were administered 6-monthly paliperidone palmitate were previously treated with the monthly and quarterly presentation of the same molecule, having presented good tolerability to it, maintaining said tolerability after treatment. change to 6-monthly paliperidone palmitate, with no adverse reaction being recorded after the change. The adherence presented by the patients was very good, performing 100% of the administrations of 6-monthly paliperidone palmitate

**Conclusions:**

6-monthly paliperidone palmitate may be an effective and well-tolerated treatment for the treatment of schizophrenia. In the present study, the use of said LAIA in a group of 40 patients is objectified, showing excellent efficacy and tolerability. All study patients were already stable with the 1-monthly and 3-monthly paliperidone palmitate formulations, maintaining said psychopathological stability when switching to the 6-monthly paliperidone palmitate formulation, with excellent adherence and adverse effect profile .

**Disclosure of Interest:**

None Declared

